# PSYCHOTHERAPY AND CARE QUALITY OF LONG-STAY NURSING HOME RESIDENTS WITH MENTAL ILLNESS OR DEMENTIA

**DOI:** 10.1093/geroni/igae098.4251

**Published:** 2024-12-31

**Authors:** Tianwen Huan, Orna Intrator, Adam Simning, Kenneth Boockvar, David C Grabowski, Shubing Cai

**Affiliations:** University of Rochester, Rochester, New York, United States; Icahn School of Medicine at Mount Sinai, New York, New York, United States; University of Rochester, Rochester, New York, United States; University of Alabama, Birmingham, Alabama, United States; Harvard Medical School, Boston, Massachusetts, United States; University of Rochester, Rochester, New York, United States

## Abstract

This study aims to fill the knowledge gap on the effect of psychotherapy on the quality of care of long-stay nursing home (NH) residents with mental illness or Alzheimer’s Disease and Related Dementias (ADRD). The 2017-2018 MDS and Medicare claims (e.g., Carrier file) and Residential History File were used to identify mental illness and ADRD diagnoses, patient characteristics, psychotherapy and quality of care. Fee-for-service Medicare beneficiaries aged 65 and older enrolled in Medicare Parts B who had six quarterly MDS assessments with/ without psychotherapy in the last four quarters were compared on two subgroups of residents: those with mental illness but no ADRD and those with ADRD. Five quarterly outcomes were examined: any 1) physical behavior; 2) verbal behavior; 3) other behavior; 4) hospitalization; 5) emergency-room visits. Covariates included predisposing, enabling characteristics and need factors. Propensity score matching was used to balance covariates between treatment and control groups. Difference-in-difference models of quarterly data, clustered within patients, were estimated for each outcome among each cohort. Analyses included 525,114/180,000 resident-quarter observations from 87,519/30,000 unique NH long-stay residents with ADRD/mental-illness-only. Among the ADRD/mental-illness-only group, 579/469 unique residents received psychotherapy. Psychotherapy was associated with lower likelihood of hospitalization (Odds ratio: 0.73, 95%CI: 0.53, 0.99) in residents with mental-illness-only. No significant results were found for the other outcomes nor the ADRD group. Findings suggest that psychotherapy may have limited effects on the quality of care for residents with ADRD but may contribute to lower hospitalization risk among residents with mental-illness-only.

